# COL3A1: Potential prognostic predictor for head and neck cancer based on immune‐microenvironment alternative splicing

**DOI:** 10.1002/cam4.5170

**Published:** 2022-08-29

**Authors:** Yuchen Shen, Xinyu Li, Deming Wang, Liming Zhang, Xiao Li, Lixin Su, Xindong Fan, Xitao Yang

**Affiliations:** ^1^ Vascular Anomaly Center, Department of Interventional Therapy, Shanghai Ninth People's Hospital Shanghai Jiao Tong University School of Medicine Shanghai China; ^2^ Shanghai Key Laboratory of Stomatology & Shanghai Research Institute of Stomatology National Clinical Research Centre for Oral Diseases Shanghai China; ^3^ Department of Neurosurgery, Shanghai Ninth People's Hospital Shanghai Jiao Tong University School of Medicine Shanghai China

**Keywords:** alternative splicing, COL3A1, HNSCC, immune checkpoint, prognosis

## Abstract

We aimed to identify a novel prognostic biomarker for head and neck squamous cell carcinoma (HNSCC) based on tumor immunology‐related alternative splicing (AS). Data for 502 HNSCC and 44 normal samples were obtained from the TCGA database and used to establish an AS‐related risk model through univariate, least absolute shrinkage, and selection operator Cox regression analyses. Fresh HNSCC and normal oral tissues were surgically obtained from 44 HNSCC patients. Western blotting and quantitative reverse transcription‐PCR were used to assess gene expression levels. Kaplan–Meier was performed to evaluate patients' overall survival (OS) rate. The CIBERSORT algorithm, single‐sample gene set enrichment analysis, and immune checkpoint analyses were performed to compare immune activities between subgroups. The risk model was established using 10 pivotal AS events first. *Collagen Type III Alpha 1 Chain* (*COL3A1*) were screened based on |log2FC| ≥ 1 and FDR < 0.05 criteria. *COL3A1* expression levels in HNSCC tissues were elevated relative to normal tissues (*p* < 0.001). Moreover, *COL3A1* was a reliable biomarker for HNSCC patients' prognostic prediction in both cohorts (*p* < 0.001, *p* = 0.0085, respectively). COL3A1 protein (*p* = 0.0054) and mRNA (*p* < 0.0001) levels were correlated with HNSCC differentiation. Furthermore, the T stage was correlated with *COL3A1* expression (*p* = 0.043), and *COL3A1* expression was an independent prognostic predictor for HNSCC patients (*p* = 0.006). Compared with the risk model, COL3A1 was better at evaluating immune cell infiltrations, immune activities, and immune checkpoint gene expressions of HNSCC lesions.

## Graphical abstract

Expression levels of COL3A1 were correlated with HNSCC differentiation but were found to be independent factors affecting HNSCC patients CloseCurlyQuote; overall survival rates. #10;COL3A1 exhibited good performance in evaluating immune cell infiltrations, immune activities, and immune checkpoint gene expressions in HNSCC.

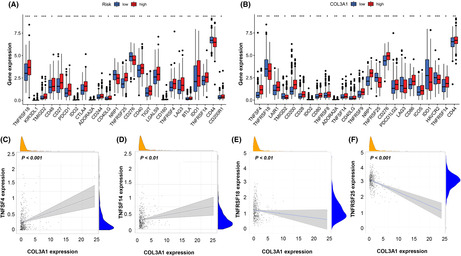

## 1 INTRODUCTION

Head and neck squamous cell carcinoma (HNSCC) is the common malignancy in the head and neck region, accounting for more than 880,000 patients/year, 7.5 billion people, and more than 450,000 deaths/year, 7.5 billion people.^1^ HNSCC patients' 5‐year survival rate remains low (50–55%).^2^

Despite high recurrence rates and low cancer survival rates, treatment advances have been made. Cancer immunotherapy, which depends on T cell–mediated immune responses, is the most significant among these advances. For instance, adoptive T cell therapy based on CART and TCR‐T has shown promising therapeutic effects in cancer patients, making it a promising strategy for cancer management.^3^ Therefore, immunotherapy is a potential approach for overcoming the limitations associated with traditional cancer therapeutic strategies.

In 2019, Li et al. presented a systematic review of immunotherapy regarding alternative mRNA splicing.^4^ Processing of pre‐mRNA transcripts is an essential step in the final function of gene products.^5^ Most human genes contain many exons, and contiguous intron sequences must be linked to pre‐transcriptional mRNA to form the mature product.^6^ AS, breaking down pre‐mRNA into a single mature transcript, contributes to the diversity of the transcriptome and proteome.^7^ Alternate acceptor site (AA), alternate donor site (AD), alternate promoter (AP), alternate terminator (AT), exon skip (ES), mutually exclusive exons (ME), and retained intron (RI) are main types.^8^ Polypeptides of tumor‐specific ribonucleic acid splicing events may bind to MHC class I (MHC‐I) molecules, which serve as a novel epitope.^9^, ^10^, ^11^ Additional analysis of the Clinical Proteomics Oncology Consortium (CPTAC) 63 breast and ovarian cancers showed that 68% of the tumors contained one or more alternatively spliced neoepitopes. In contrast, only 30% of tumors contained neoepitopes derived from somatic events with single‐nucleotide variants.^4^ These findings form the basis for studies on immune‐related AS events in tumors.

Despite several promising risk models of HNSCC based on AS,^12^, ^13^, ^14^ deficiencies in these models exist, and the correlation between AS and tumor immunology in HNSCC has yet to be elucidated. Therefore, a systematic analysis of HNSCC‐related AS events should be performed from a tumor immune microenvironment (TIM) perspective. In this study, we aimed at i. establishing a risk model for HNSCC patients based on pivotal AS events; ii. screening genes can be used as prognostic predictors for HNSCC patients; and iii. we are evaluating the accuracy of the risk‐model and prognostic gene according to the status of the TIM.

## 2 MATERIALS AND METHODS

### 2.1 Data acquisition for alternative splicing events

RNA sequencing data for 502 HNSCC and 44 normal samples were retrieved from The Cancer Genome Atlas (TCGA) and the HNSCC cohort (https://tcga‐data.nci.nih.gov/tcga/). SpliceSeq was used to assess mRNA splicing patterns of samples. Percent Spliced In (PSI) values, ranging from zero to one, which is commonly used in quantifying AS events, were imputed for seven different AS events.^15^ AS events with PSI values >75% were included. After screening HNSCC patients, we finally included 487 HNSCC samples and 44 normal controls. All clinicopathological data were obtained from TCGA. Immunohistochemical (IHC) data were downloaded from Proteinatlas (http://www.proteinatlas.org/pathology) to assess the expressions of differentially expressed genes in HNSCC.

### 2.2 Patients and sample collection

Forty‐four patients' fresh HNSCC and their healthy tissues were collected, all of which were resected during surgery in the Department of Oral and Maxillofacial‐Head and Neck Oncology, Shanghai Ninth People's Hospital from April 2010 to October 2014.^16^ The patients including 25 males and 19 females, aging from 35 to 86, with an average of 59 ± 12.4. For tissue collection, the method was the same as our previous study.^17^ Subsequently, the pathological diagnosis was carried out in the Department of Pathology, Shanghai Ninth Public Hospital. The clinical evaluation of all cases was carried out according to the 8th edition of the TNM classification of head and neck cancer.^18^ Based on pathological reports, among the 44 cases, 15 cases were characterized as high‐differentiation HNSCC, 20 cases were characterized as moderate‐differentiation HNSCC, and 9 cases were characterized as low‐differentiation HNSCC. A telephone survey followed up all 44 HNSCC patients until October 2019.

### 2.3 Identification of survival‐related alternative splicing events and construction of the prognostic model

We performed a univariate Cox regression analysis to identify AS events related to overall survival (OS) with *p* < 0.05. Upset plots were generated using UpsetR for quantitative analysis of interaction sets among the seven types of OS‐related AS. We selected the highest AS events associated with survival as candidates for each splice type to fit Least absolute shrinkage and selection operator (LASSO) Cox analysis for HNSCC. A risk score was calculated for each prognostic model from the sum of the PSI values of the identified AS events and the corresponding coefficients derived from multivariate Cox analysis. The formula for calculating the risk score was as previously described^17^:

$$\text{Risk score}=\sum_{i}^{n}\beta_{i}\times {\mathrm{PSI}}_{i}$$

N represents the quantity of AS events, β are the coefficients, while PSI denotes PSI values for specific AS events.

### 2.4 Identification of differentially expressed genes

Before comparisons, we normalized the expression data in the TCGA database as fragment per kilobase million (FPKM) values. The “limma” package was used to identify differentially expressed genes (DEGs) using |log2FC| ≥ 1 and FDR <0.05 as the cut‐offs. Moreover, based on each DEG expression level, we equally divided the TCGA cohort into low‐ and high‐expression subgroups.

### 2.5 Immune function analyses

Estimation of Stromal and Immune cells in Malignant Tumor tissues using Expression data (ESTIMATE) database (https://bioinformatics.mdanderson.org/public‐software/estimate/) was used to generate immune score and stromal score, which represent the degrees of infiltrations of immune and stromal cells in tumor tissues, respectively. Data on tumor‐infiltrating immune cells were estimated using the CIBERSORT algorithm. The “gsva” package was used to perform a single‐sample gene set enrichment analysis (ssGSEA) and to calculate the infiltrating immune cells' scores and responses to assess the activities of immune pathways.

### 2.6 Protein extraction and western immunoblotting

For western immunoblotting (WB), we referred to our previous study for the relevant operating steps.^2^ Primary antibodies against COL3A1 (ab184993, 1:1000 dilution; Abcam [UK]), GAPDH (WL01547, 1:1500 dilution; Wanlei Bio), and the secondary goat‐anti‐rabbit antibody (ZB‐2301; Zhong Shan Golden Bridge) were used in this study. WB bands were detected by Alpha FluorChem FC3 (Protein Simple), whereas imaging data were quantified by the ImageJ software as previously mentioned.^2^

### 2.7 Total RNA extraction, reverse transcription, and qRT‐PCR

Total RNA was extracted from clinical specimens using RNAiso Plus (9109, TAKARA), after which cDNA was synthesized from 10 μg of the extracted total mRNA using the PrimeScript™RT Master Mix (RR036A, TAKARA), according to the manufacturer's instructions. Quantitative reverse transcription‐PCR (qRT‐PCR) was performed using Power SYBR Green PCR Master Mix (4,367,659, Thermo Fisher Scientific) and Q2000B (LongGene). Primers for COL3A1 and GAPDH were obtained with reference to PrimerBank (https://pga.mgh.harvard.edu/primerbank/index.html) (Table S1). Relative differences in expression between samples were analyzed by the 2^−ΔΔCt^ method. GAPDH was selected as an internal reference.

### 2.8 Immune checkpoint analysis based on the risk model

We screened and extracted 46 immune checkpoint genes (ICGs) from previous studies^19^, ^20^, ^21^, ^22^ (Table S2). Then, gene expression data for the TCGA cohort was used to analyze the correlation between risk score and each ICG. Second, the data was used to assess the expression profiles of ICGs between subgroups. Both processes were performed using R.

### 2.9 Statistical analysis

R (v4.0.2), SPSS 24.0, and Prism Graphpad 8 were used for statistical analysis. Two‐tailed students' t‐tests and one‐way ANOVA were used to analyze the correlation between groups. The Kaplan–Meier analysis evaluated the OS rate, and the two‐tailed log‐rank test and Mann–Whitney U‐test were used to compare the differences between the two groups. Univariate and multivariate Cox regression analyses were carried out to calculate the survival hazard ratio (HR) and 95% confidence interval (CI). *p* value <0.05 was considered statistically significant.

## 3 RESULTS

### 3.1 Overview of alternative splicing event profiles and construction of the prognostic risk model in head and neck squamous cell carcinoma

**FIGURE 1:**
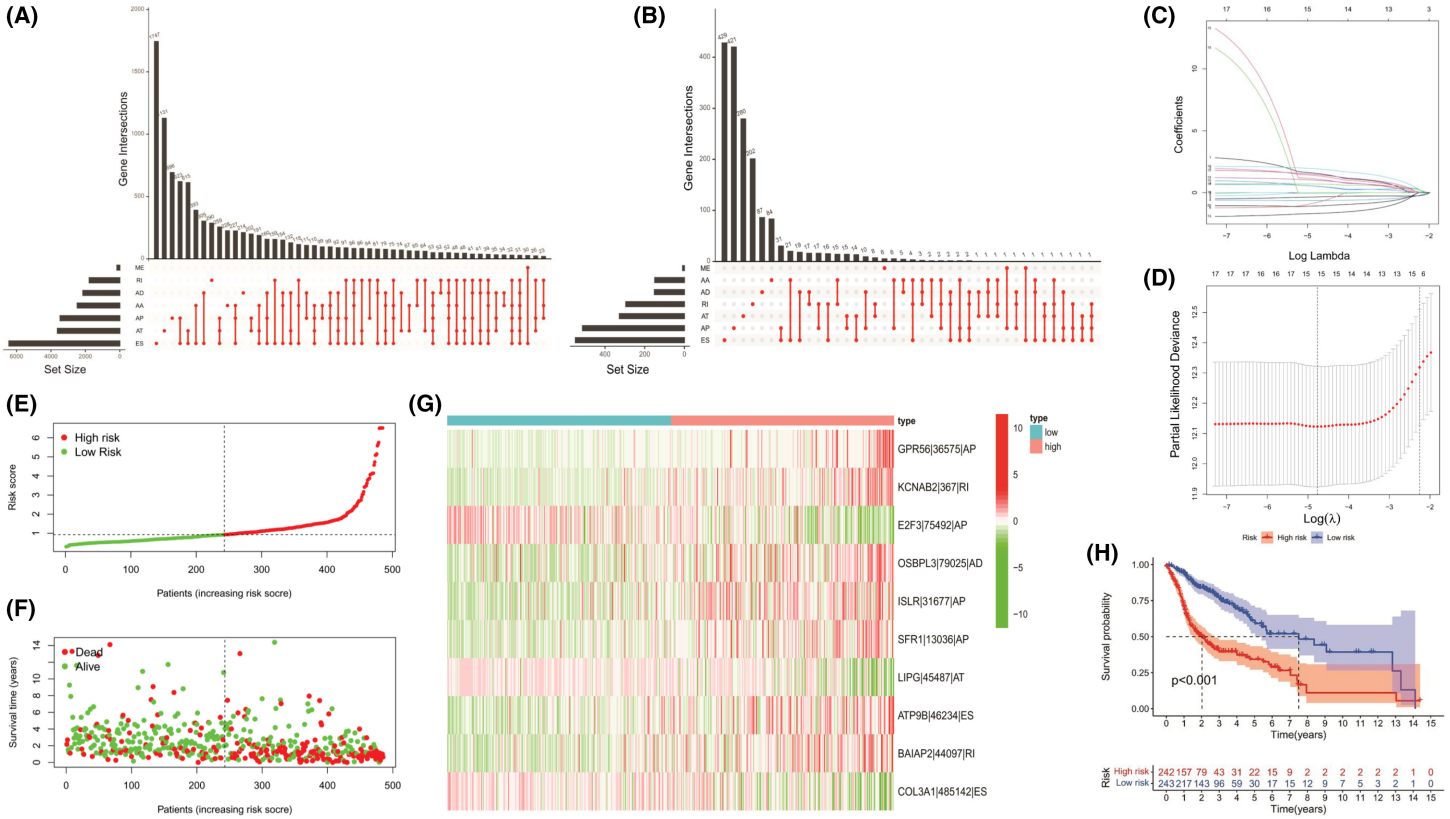
Overall review of the risk model's alternative splicing (AS) events and construction processes. (A) Upset plot of interaction among seven standard AS events in head and neck squamous cell carcinoma (HNSCC). (B) Upset plot of interactions among the seven survival‐associated AS events in HNSCC. (C) LASSO regression of survival‐associated AS events. (D) Cross‐validation to optimize parameter selection in LASSO regression. (E) Distribution of patients based on the risk score. (F) All patients' survival status (low‐risk population: dotted line on the left side; high‐risk population: dotted line on the right side). (G) Heatmap (green: low PSI value; red: high PSI value) of the 10 pivotal AS events between the low‐risk group (brilliant blue) and the high‐risk group (red). (H) Kaplan–Meier curve between the low‐risk group and the high‐risk group.

First, UpSet plot were generated to visualize interactive sets between the seven types of AS events. Figure 1A,B showed that a total of 42,849 AS events were detected from 10,123 genes. Univariate Cox analysis found 2893 survival‐related AS events within 1733 genes in our HNSCC cohort (all *p* < 0.05). For each AS type, the top 20 survival‐related events in HNSCC are shown in Figure S1 (only 8 for ME). Then, we performed a LASSO Cox analysis for HNSCC, and 10 pivotal AS events for establishing the risk model were screened (Figure 1C,D, Table S3). After multivariate Cox analysis had been performed, our risk‐score formula was as follows:

$$\begin{array}{c} \text{Risk score}=\left(2.19^{*}{\mathrm{PSI}}_{\mathrm{GPR}56|36575|\mathrm{AP}}\right)+\left(1.80^{*}{\mathrm{PSI}}_{\text{KCNAB}2|367|\mathrm{RI}}\right)+\left(-0.78^{*}{\mathrm{PSI}}_{E2F3|75492|\mathrm{AP}}\right)+\\ \left(-1.13^{*}{\mathrm{PSI}}_{\text{OSBPL}3|79025|\mathrm{AD}}\right)+\left(0.91^{*}\;{\mathrm{PSI}}_{\text{ISLR}|31677|\mathrm{AP}}\right)+\left(1.70^{*}\;{\mathrm{PSI}}_{\mathrm{SFR}1|13036|\mathrm{AP}}\right)+\\ \left(-1.92^{*}{\mathrm{PSI}}_{\text{LIPG}|45487|\mathrm{AT}}\right)+\left(2.23^{*}{\mathrm{PSI}}_{\mathrm{ATP}9B|46234|\mathrm{ES}}\right)+\left(1.24^{*}{\mathrm{PSI}}_{\text{BAIAP}2|44097|\mathrm{RI}}\right)+\left(-1.93^{*}{\mathrm{PSI}}_{\mathrm{COL}3A1|485142|\mathrm{ES}}\right). \end{array}$$

HNSCC patients were equally divided into low‐ and high‐risk subgroups based on the median score (Figure 1E). Compared with patients in the low‐risk group, patients in the high‐risk group exhibited higher death rates and shorter survival times (Figure 1F). The heat map presents the overall situation of the 10 fundamental AS events, as shown in Figure 1G.

Kaplan–Meier analysis showed that the patients in the low‐risk group enjoyed better prognostic outcomes relative to their counterparts (*p* < 0.001, Figure 1H). Univariate and multivariate analyses suggested that the risk score could be an independent factor influencing HNSCC patients' prognoses (*p* < 0.001, Figure S2A). The ROC curves showed that the prediction model based on risk scores had better differentiating abilities (AUC for risk score = 0.763, Figure S2B; AUC at 1 year = 0.763, AUC at 2 years = 0.756; AUC at 3 years = 0.745, Figure S2C).

### 3.2 Comparisons of immune activities between the risk‐subgroups

**FIGURE 2:**
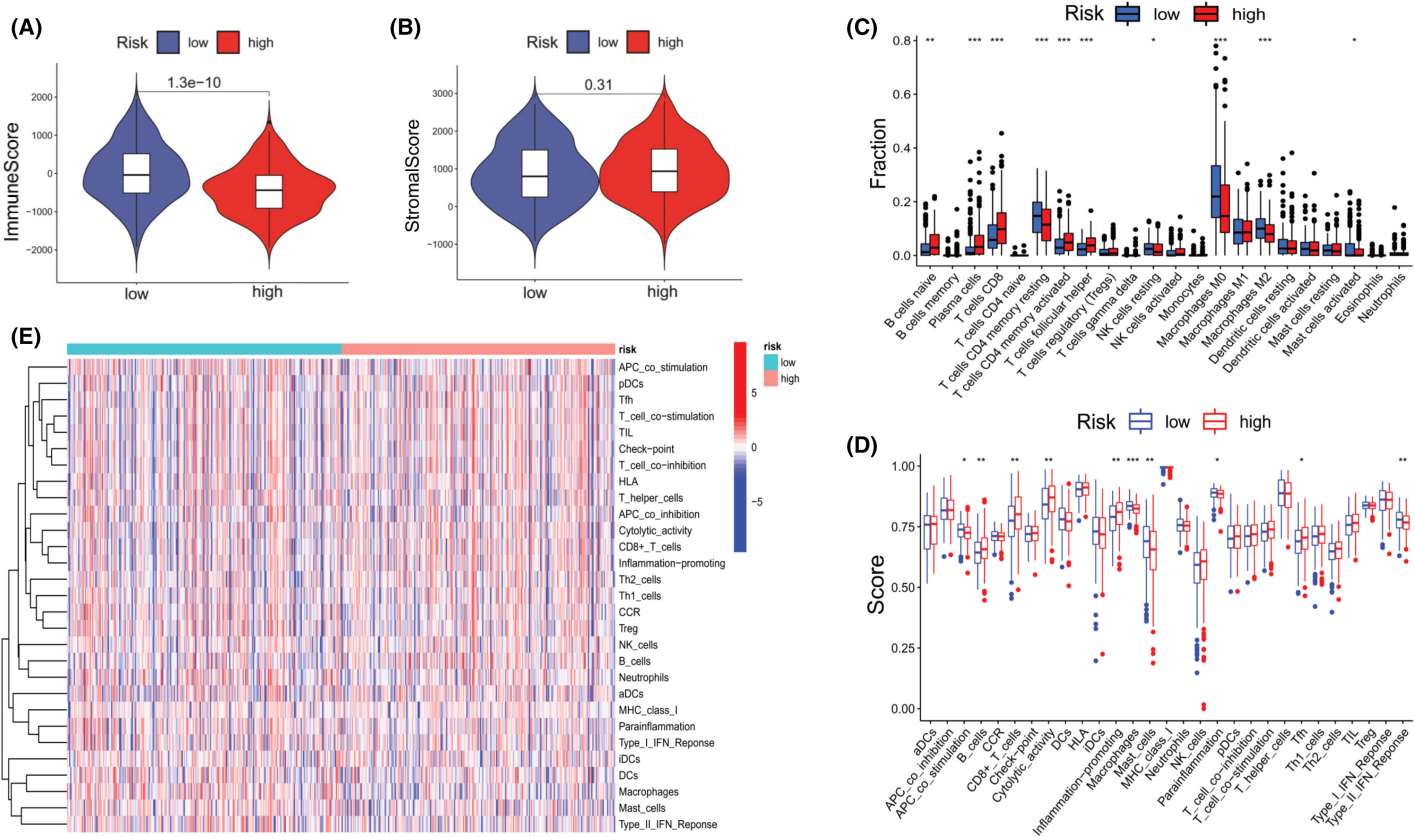
Immune activities of the low‐ and high‐risk groups. (A) Immune scores for both subgroups. (B) Stromal scores for both subgroups. (C) Immune cell infiltrations in both subgroups. (D) Scores of immune activities for both subgroups. (E) Overall heatmap of immune cells and responses (red: high expression; blue: low expression) of pyroptosis genes between the low‐risk (brilliant blue) and high‐risk (red) groups. (Two‐tailed students' *t*‐test, *p < 0.05; **p < 0.01; ***p < 0.001).

Then, we used the ESTIMATE database to assess the status of each HNSCC patient's TIM. As shown in Figure 2A, patients from the low‐risk group received higher immune scores compared with the high‐risk group (*p* < 0.0001); however, no significant differences were found between the two subgroups with regard to stromal scores (*p* = 0.31, Figure 2B). These findings indicate that low‐risk patients had higher infiltration rates of immune cells and more active immune responses in the tumor microenvironment than the high‐risk patients. For immune cells, low‐risk patients' resting CD4^+^ T cells, M0, and M2 macrophages were significantly increased compared with high‐risk patients (all *p* < 0.001, Figure 2C). With regard to immune score, low‐risk patients exhibited more infiltration of macrophages, mast cells and type II IFN responses, relative to high‐risk patients (*p* < 0.001, *p* < 0.01, *p* < 0.01, Figure 2D,E).

### 3.3 Identification of differentially expressed gene and immune‐related analysis

**FIGURE 3:**
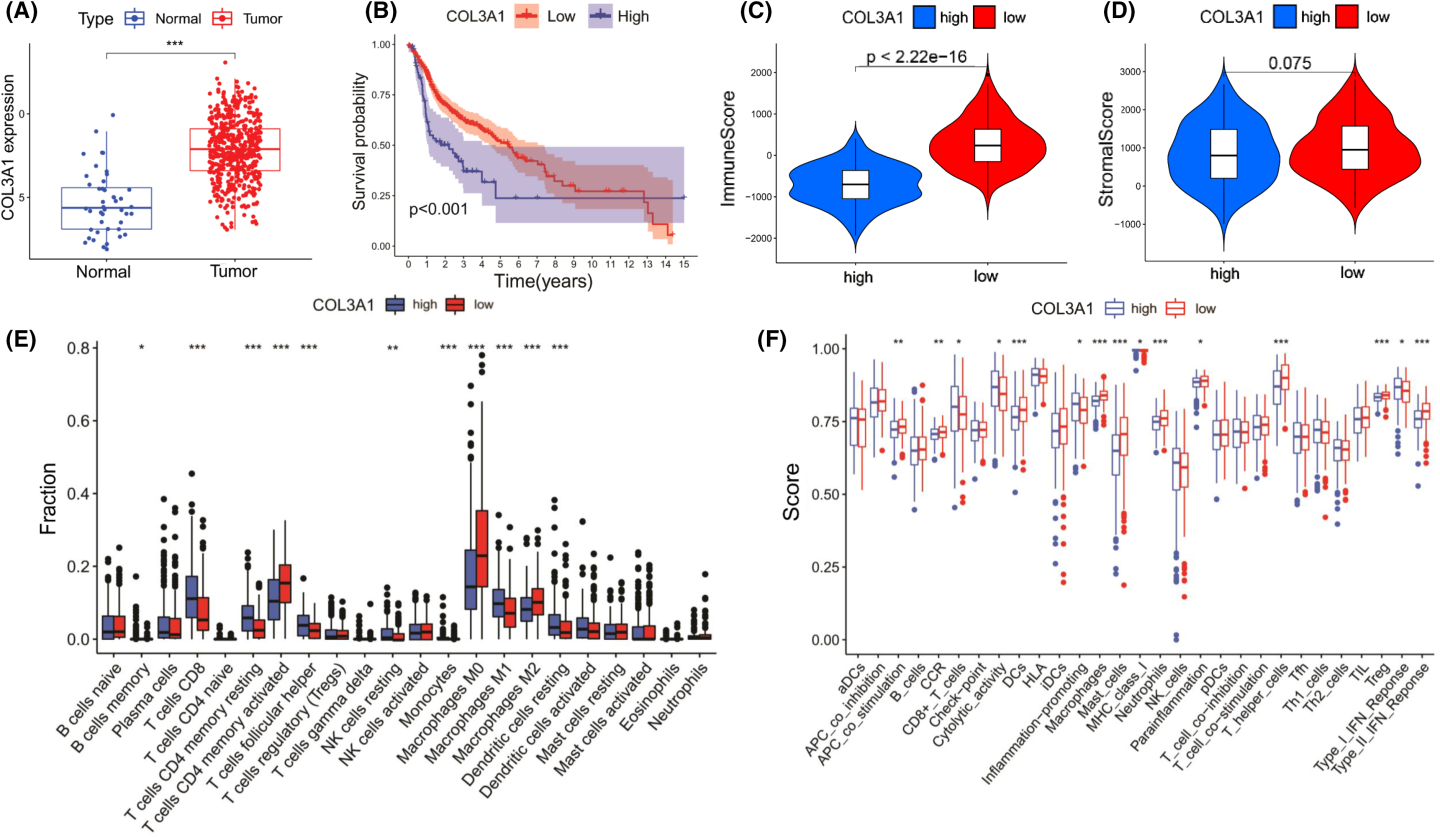
Prognosis and immune status between head and neck squamous cell carcinoma (HNSCC) patients with different *COL3A1* expression levels. (A) *COL3A1* expression levels between HNSCC and normal tissues. (B) Kaplan–Meier analysis between *COL3A1* low‐ and high‐expression groups. (C) Immune scores for both subgroups. (D) Stromal scores for both subgroups. (E) Immune cell infiltrations in both subgroups. (F) Scores of immune activities for both subgroups. (Two‐tailed students' *t*‐test, *p < 0.05; **p < 0.01; ***p < 0.001).

Next, we screened the TCGA cohort's DEGs from the risk model's 10 pivotal AS events. Only *Collagen Type III Alpha 1 Chain* (*COL3A1*) met the |log2FC| ≥ 1 and FDR <0.05 criteria (data not shown). HNSCC patients from the TCGA cohort exhibited significant differences in *COL3A1* expression levels compared with normal patients (*p* < 0.001, Figure 3A). Then, we equally assigned the HNSCC patients into two subgroups according to their *COL3A1* levels. Notably, Kaplan–Meier analysis of OS based on *COL3A1* expression levels indicated that *COL3A1* could be used for prognostic prediction of HNSCC patients. HNSCC patients with suppressed *COL3A1* expression levels were associated with high OS rates (*p* < 0.001, Figure 3B). Since AS can affect HNSCC patients' TIM, then, based on the ESTIMATE database, we evaluated whether *COL3A1* has the same effects. Interestingly, the group with low *COL3A1* expression had a higher immune score (*p* < 0.0001) but not stromal score (*p* = 0.075), relative to the high‐expression group, suggesting that HNSCC patients with low COL3A1 levels had more activated immune status in TIM than their counterparts (Figure 3C,D). With regard to immune cells, patients with elevated COL3A1 levels had more resting CD4^+^ T cells and resting natural killer (NK) cells but less activated CD4^+^ T cells compared with *COL3A1* low‐expressing patients (all *p* < 0.001, Figure 3E). Patients with elevated COL3A1 levels were generally associated with less immune activity than those with suppressed levels (Figure 3F).

### 3.4 Correlations between *COL3A1* expressions and survival outcomes for head and neck squamous cell carcinoma patients

**FIGURE 4:**
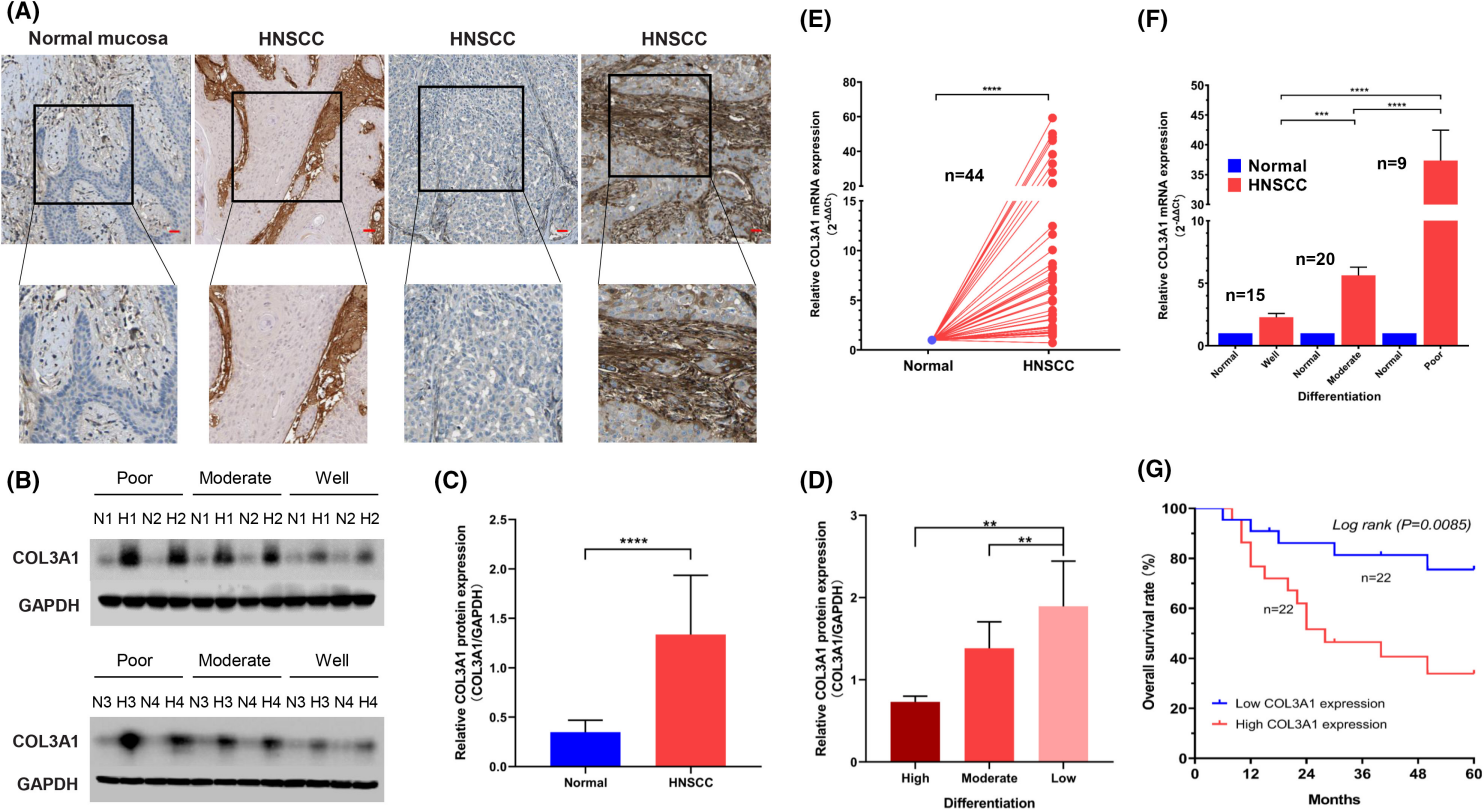
Comparisons of *COL3A1* expressions between head and neck squamous cell carcinoma (HNSCC) and normal tissues and survival outcomes. (A) Representative immunohistochemical images of paraffin‐embedded HNSCC and normal oral samples (Bar: 50 μm). (B) Representative WB images for four patients from each HNSCC differentiation. (C) Relative COL3A1 protein levels in HNSCC and normal tissues as evaluated by band intensities and areas in WB (*n* = 44). (D) Comparisons of COL3A1 protein levels among differentially differentiated HNSCC tissues (*p* = 0.0054). (E) Comparisons of relative *COL3A1* mRNA levels between 44 HNSCC and 44 normal control tissues. (F) Comparisons of relative *COL3A1* mRNA levels among each HNSCC differentiation (*p* < 0.0001). (G) Kaplan–Meier curves for 44 HNSCC patients based on their *COL3A1* mRNA levels. (One‐way ANOVA and two‐tailed students' *t*‐test, **p < 0.01; ***p < 0.001; ****p < 0.0001).

To analyze the expression levels of *COL3A1* in HNSCC, first, we assessed overall differences between paraffin‐embedded HNSCC samples and normal oral samples using the IHC data in Proteinatlas. Protein levels of COL3A1 in HNSCC samples were higher than those of the normal oral mucosa samples (Figure 4A). Next, we performed WB to assess protein levels of COL3A1 in fresh tissues of practical HNSCC patients. Figure 4B,C showed that COL3A1 levels were about threefold higher in fresh HNSCC compared with normal oral tissues (*p* < 0.0001). Interestingly, protein levels of COL3A1 significantly increased as histopathologic grades for HNSCC decreased (Figure 4D, *p* < 0.01).

Next, we extracted total mRNA from sections of HNSCC and normal control tissues and assessed mRNA levels of *COL3A1*. *COL3A1* mRNA expressions were elevated in HNSCC tissues compared with normal control tissues (Figure 4E, *p* < 0.001). Surprisingly, as shown in Figure 4F, though the general tendency of *COL3A1* mRNA levels among each HNSCC differentiated tissue was close to WB results, *COL3A1* mRNA levels in poorly differentiated HNSCC tissues were nearly 50‐fold compared with normal tissues. Then, we equally assigned the 44 HNSCC patients into two subgroups according to their *COL3A1* mRNA levels and performed Kaplan–Meier analyses to compare OS rates between the two groups. As shown in Figure 4G, the cumulative five‐year OS rate was 40.9% (18 of 44). Kaplan–Meier analysis demonstrated that OS outcomes for patients with elevated *COL3A1* levels were significantly low relative to those with suppressed *COL3A1* levels (*p* = 0.0085).

### 3.5 Correlations between *COL3A1* levels and head and neck squamous cell carcinoma patients' clinicopathological features

Correlations between *COL3A1* expression levels and potential risk factors (smoking and alcohol histories) for 44 HNSCC patients and the association between *COL3A1* levels, sex, and age (divided by 60 years) were analyzed. Among the 44 HNSCC patients, 15 cases were high differentiation, 20 were moderate differentiation, and 9 were low differentiation. These patients were classified into the high *COL3A1* expressing group (22 cases) and the low *COL3A1* expressing group (22 cases). As Table 1 showed, expression levels of *COL3A1* were significantly correlated with the T stage (*p* = 0.043). *COL3A1* expression levels were not significantly different from clinical parameters such as sex, age, smoking status, alcohol history, clinical status, and lymph node metastasis.

**TABLE 1:** Correlation between the expression of COL3A1 and patients' case history and clinicopathological characteristics

Characteristics	Number of cases (%)	COL3A1 expression		*p* ^a^ value
		High (%)	Low (%)	
Sex				0.373
Male	25 (56.8)	11 (25.0)	14 (31.8)	
Female	19 (43.2)	11 (25.0)	8 (18.2)	
Age				0.373
≤60	25 (56.8)	14 (31.8)	11 (25.0)	
>60	19 (43.2)	8 (18.2)	11 (25.0)	
Smoking history				0.757
Present	29 (65.9)	14 (31.8)	15 (34.1)	
Absent	15 (34.1)	8 (18.2)	7 (15.9)	
Alcohol history				0.764
Present	27 (61.4)	13 (29.5)	14 (31.8)	
Absent	17 (38.6)	9 (20.5)	8 (18.2)	
T stage				**0.043** *
T1 + T2	32 (72.7)	13 (29.5)	19 (43.2)	
T3 + T4	12 (27.3)	9 (20.5)	3 (6.8)	
Clinical stage				0.062
I‐II	28 (63.6)	11 (25.0)	17 (38.6)	
III‐IV	16 (36.4)	11 (25.0)	5 (11.4)	
Lymphatic metastasis				0.365
Positive	17 (38.6)	10 (22.7)	7 (15.9)	
Negative	27 (61.4)	12 (27.3)	15 (34.1)	

Bold value indicate Statistical significance.

^a^ All statistical tests were two‐sided.

* *p* < 0.05.

### 3.6 Univariate and multivariate analysis of head and neck squamous cell carcinoma patients' prognoses

Univariate Cox models were used to estimate individual clinical parameters for OS to establish the potential prognostic value. As shown in Table 2, significant factors for OS were T stage (*p* < 0.01), clinical tumor stage (*p* = 0.048 < 0.05) and *COL3A1* expression levels (*p* < 0.01). Subsequently, these factors were included in the multivariate Cox regression model, which further showed that *COL3A1* expression was the independent factor for OS (*p* = 0.006, HR = 3.731, 95% CI: 1.458–9.546).

**TABLE 2:** Univariate and multivariate analyses of clinical characteristics for overall survival in patients with head and neck squamous cell carcinoma

Characteristics	Amount (%)	Univariate		Multivariate	
		*p* ^a^ value	HR^b^ (95% CI^c^)	*p* ^a^ value	HR^b^ (95% CI^c^)
Age					
≤60	25 (56.8)	0.321	1.580 (0.612–4.078)		
>60	19 (43.2)				
Sex					
Male	25 (56.8)	0.706	0.835 (0.329–2.123)		
Female	19 (43.2)				
Smoking history					
Present	29 (65.9)	0.102	2.111 (0.750–5.942)		
Absent	15 (34.1)				
Alcohol history					
Present	27 (61.4)	0.228	0.540 (0.212–1.378)		
Absent	17 (38.6)				
T stage					
T1 and T2	32 (72.7)	**<0.01** **	3.609 (1.177–11.07)	0.073	
T3 and T4	12 (27.3)				
Clinical stage					
Stage I and II	28 (63.6)	**0.048** *	2.450 (0.922–6.506)	0.798	
Stage III and IV	16 (36.4)				
Lymphatic metastasis					
Positive	17 (38.6)	0.105	0.478 (0.184–1.241)		
Negative	27 (61.4)				
COL3A1 expression					
Low	22 (50)	**<0.01** **	1.275 (1.118–1.358)	**0.006** **	3.731 (1.458–9.546)
High	22 (50)				

Bold values indicate Statistical significance.

^a^ All statistical tests were two‐sided.

^b^ HR, Hazard ratio.

^c^ CI, confidence interval.

* *p* < 0.05

** *p* < 0.01.

### 3.7 Correlation between immune checkpoint genes and the prognostic model or *COL3A1* expression

**FIGURE 5:**
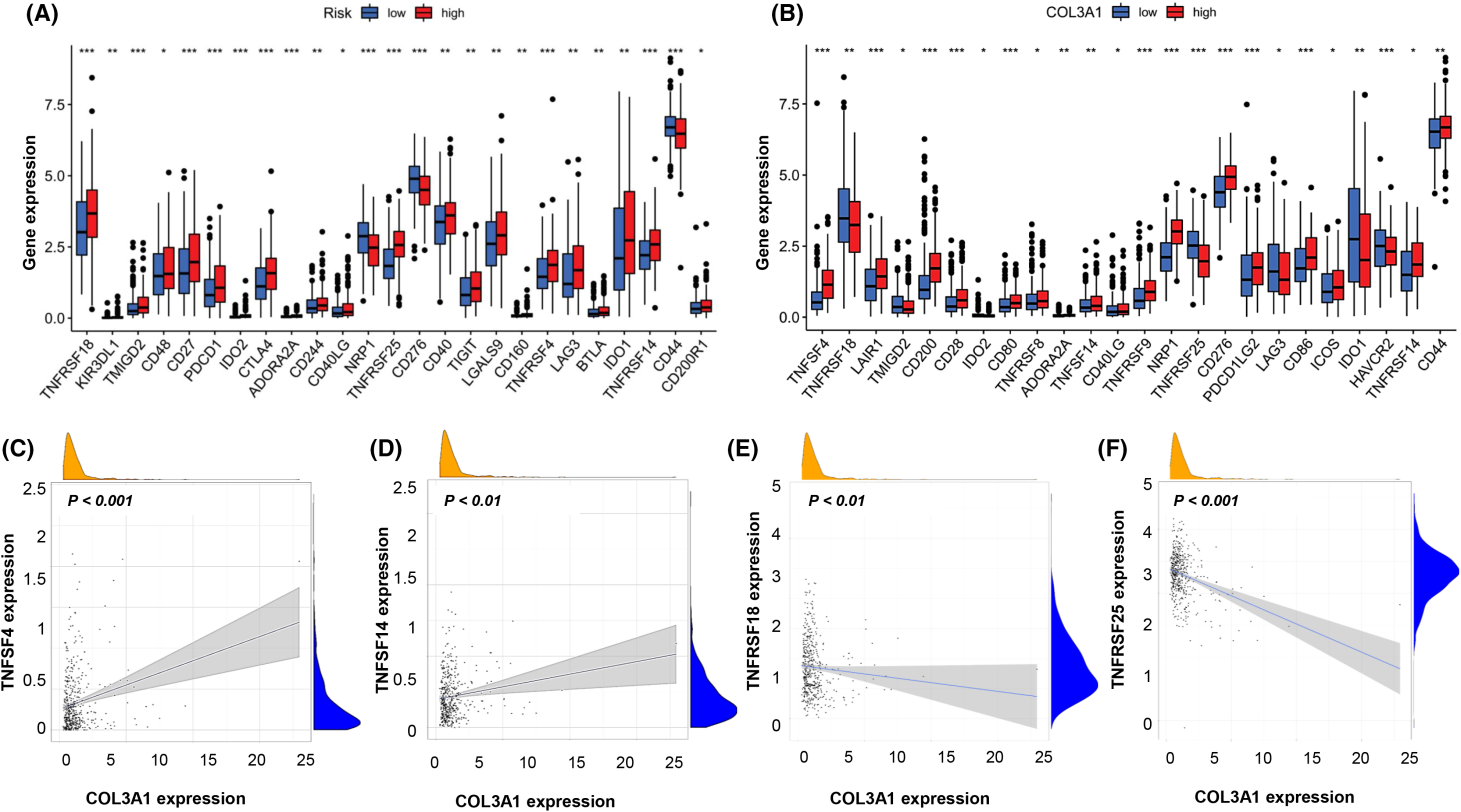
Correlation between immune checkpoint genes (ICGs) and *COL3A1*. (A) Differences in ICGs levels between low‐ and high‐risk groups. (B) Differences in ICG levels between *COL3A1* low‐ and high‐expression groups. (B‐F) The correlations between gene expression levels of *TNFSF4*, *TNFSF14*, *TNFRSF18*, *TNFRSF25*, and *COL3A1* expression. (Two‐tailed students' *t*‐test, *p < 0.05; **p < 0.01; ***p < 0.001).

Given the important roles of immune checkpoints in tumor immune escape, we determined differences in expressions for each of the ICGs between low‐ and high‐risk groups. These ICGs include TNFRSF18, TMIGD2, CD27, PD1, IDO2, CTLA4, ADORA2A, NRP1, TNFRSF25, CD276, TNFRSF4, TNFRSF14, and CD44 exhibited the highest differences between the two subgroups (Figure 5A, all *p* < 0.001). Next, we evaluated the tendency of gene expression between ICGs and the *COL3A1*. As shown in Figure 5B, patients from *COL3A1* low‐ and high‐expression groups were endowed with significant differences in gene expression status of ICGs. Between the subgroups, *TNFSF4*, *LAIR1*, *CD200*, *CD28*, *CD80*, *TNFRSF9*, *NRP1*, *TNFRSF25*, *CD26*, *PD1LG2*, *CD86*, and *HAVCR2* were differentially expressed (all *p* < 0.001). Furthermore, we found that superfamilies of TNF and their receptors, namely TNFSF and TNFRSF, were dominant among ICGs. The expression of *TNFSF4*, *TNFSF14*, *TNFRSF18*, and *TNFRSF25* in HNSCC patients and their correlation with COL3A1 were detailed in Figure 5C‐F (all *p* < 0.05).

## 4 DISCUSSION

AS is a common gene expression regulation mechanism that allows genes to produce several different types of mRNA. AS is tightly regulated in different tissues, cell types, and stages of differentiation.^23^, ^24^, ^25^ In many malignancies, AS is essential in tumorigenesis.^26^, ^27^, ^28^, ^29^ Kahles et al. found that healthy tissue bears fewer AS events than tumors.^9^ Moreover, the impact of AS on the immune system is a potential predictor for responses to immune checkpoint‐related therapy. Cascades of immune checkpoints, including those controlled by programmed cell death 1 (PD‐1) or T‐cell cytotoxic antigen 4 (CTLA‐4), could be treated as negative regulators of immunity. Therefore, suppressing these cascades with antibodies has transformed many cancers' treatment, leading to high levels of enduring tumor responses.^30^, ^31^, ^32^ In HNSCC, studies should evaluate how AS can benefit prevention, diagnosis, treatment, and prognostic prediction from a tumor immune perspective.

In this study, we first established a prognostic risk model based on AS events in HNSCC patients. Ten events were screened and included in the risk formula. Even though our risk model was shown to have the ability for prognostic prediction, there were some limitations when assessing HNSCC patients' immune statuses. For instance, as shown in Figure 2C, low‐risk patients exhibited high infiltration levels of resting CD4^+^ T cells and resting NK cells but less activated CD4^+^ T cells relative to high‐risk patients. These findings are inconsistent with natural immune microenvironment conditions for patients with high‐grade HNSCC.^33^, ^34^ Therefore, the risk model must refine the key genes to enhance its predictive accuracy.

Collagen Type III Alpha 1 Chain, also known as Collagen Alpha‐1(III) Chain, is a protein encoded by the *COL3A1* gene in humans. Type III collagen is involved in many cellular functions despite its interaction with integrins and cell surface receptors.^35^ As shown in Figure 3E,F, regarding the TIM of HNSCC, COL3A1 exhibited better performance than the risk model (Figures C and D). Even though the risk model and *COL3A1* could be used to predict HNSCC patients' prognoses, the latter exhibited a better ability to predict HNSCC patients' tumor immune responses.

COL3A1 has been shown to promote neoplasia in several types of malignancies^36^, ^37^; however, its role in HNSCC evolution has not been conclusively determined. Our study demonstrated that COL3A1 exhibited the potency to become a prognostic biomarker for HNSCC, for it was more activated in cancer tissues than normal tissues, not only in the TCGA cohort but also in the practical patient cohort. Elevated *COL3A1* levels were associated with poor prognostic outcomes for HNSCC patients, and as the differentiation of HNSCC tissues improved, COL3A1 expression levels reduced. For patients' case history and clinicopathological characteristics, only the T stage was correlated with COL3A1 (Table 1). Through univariate and multivariate analyses, COL3A1 expression was an independent factor in predicting the HNSCC patient's OS rates (Table 2).

The present study found that COL3A1|485,142|ES is a critical AS event as it exhibited a relatively high absolute coefficient value in the formula. Per a former report, the most frequent pattern of abnormal splicing of COL3A1 is exon skipping (ES).^38^ COL3A1 plays a pivotal role in the extracellular matrix. It has been reported that the abnormal expression of *COL3A1* was closely associated with fibrosis in the cardiovascular system and spinal ligaments, indicating COL3A1’s capacity to maintain the functions of fibroblasts.^39^, ^40^ Rachel et al. found that patients with vascular Ehlers‐Danlos syndrome, a genetic disease caused by the *COL3A1* exon skipping mutations and presented with increased vascular fragility, suffered from systemic inflammation.

Given the above findings, we evaluated the underlying immune mechanisms. Cancer cells can prevent immune surveillance and progression through a series of mechanisms, including activation of immune checkpoint pathways to suppress immune responses against cancer.^41^ Tumor immunotherapy based on immune‐checkpoint blockade is a novel strategy for coping with refractory neoplasms. By blocking negative regulators of the innate immunity such as cytotoxic T lymphocyte antigen 4 (CTLA‐4) and programmed cell death 1 (PD‐1) or their ligands, programmed cell death ligand 1 (PD‐1), immune checkpoint blockade enhances antitumor immunity.^42^ Due to the potential effects of AS events on TIM via affecting ICGs, we evaluated the correlation between ICGs and our risk model or *COL3A1* expression. As shown in Figure 5A,B, high‐risk group patients exhibited more ICGs activities, implying that cancer cells in high‐risk patients' HNSCC lesions have an enhanced ability to escape supervisory control and the threat from the immune system. These outcomes were also found in HNSCC patients whose *COL3A1* expression levels were higher, which could explain why both high‐risk HNSCC patients and high‐*COL3A1* expression patients are prone to poor prognostic outcomes.

Another interesting finding was that genes of the *TNFSF* and *TNFRSF* were differentially expressed between the high‐ and low‐risk subgroups and high‐ and low‐*COL3A1* expression groups (Figure 5A,B). The above has revealed the potential role of *TNFSF* and *TNFRSF* members in facilitating HNSCC progression and, therefore, as potential targets for HNSCC immunotherapy. First, *TNFRSF25* was differentially expressed between low‐ and high‐*COL3A1* expression subgroups (Figure 5E). *TNFRSF25 is* mainly expressed in activated T cells, antigen‐presenting cells (APCs), and phagocytes.^43^, ^44^, ^45^ Activation of TNFRSF25 by its ligand, TNFSF15, is important for T‐cell proliferation during viral infection control as it enhances interferon‐g (IFN‐g) synthesis.^46^ Co‐stimulatory *TNFRSF25* promotes the differentiation of Th1 and Th9 CD4^+^ T cells. It dampens the suppressive capacity of T_reg_ cells, implying that an agonistic antibody for TNFRSF25 is an attractive agent for cancer immunotherapy.^47^, ^48^ In contrast to *COL3A1* expression, the risk model did not represent the actual immue situation of HNSCC lesions as high‐risk patients exhibited elevated *TNFRSF25* levels relative to low‐risk patients.

A similar advantage of COL3A1 was also confirmed by *TNFRSF18* expression (Figure 5F). *TNFRSF18* is mainly expressed in APCs and medullary thymic epithelial cells. Furthermore, *TNFRSF18* is highly expressed in T_reg_ progenitors, and activating ligands may promote its development in mature thymic T_reg_ cells by upregulating CD25.^49^, ^50^ The binding of TNFRSF18 to its ligand, TNFSF18, can suppress T_reg_ cell recruitment, weaken their inhibitory functions and activate the mitogen‐activated protein kinase (MAPK)/ extracellular regulated protein kinases (ERK) pathway as well as nuclear factor kappa B (NF‐κB) signaling, thereby promoting T‐cell proliferation, secretion of pro‐inflammatory cytokines, and enhancing antitumor functions.^51^ Previous findings from studies involving the agonistic TNFRSF18 antibody, TRX518, laid the groundwork for clinical trials in malignant melanoma or other solid cancers.^52^

Finally, it was also noteworthy that patients with elevated COL3A1 levels had a higher level of resting CD4^+^T cells and resting NK cells, but fewer activated CD4+ T cells than their counterparts (Figure 3E,F). Differences in *TNFSF* and *TNFRSF* gene expressions might shed light on these intriguing results. According to the previous studies, *TNFRSF18* is weakly expressed by naive CD4^+^T cells but is upregulated on activation. In comparison, *TNFRSF25* is upregulated in activated CD4^+^T cells, B cells, NK cells, and NKT cells.^53^ Thus, our results in Figure 5E,F consolidated the standpoint that HNSCC patients with higher *COL3A1* expression generally possessed a relatively suppressed condition of immune cells owing to low‐level *TNFRSF18* and *TNFRSF25*.

Although we found that COL3A1 has the potential for prognostic prediction of HNSCC, a total of 44 HNSCC patients and their control samples represent relatively small sample size. Additionally, immune cells can intimately interact with stromal cells and further impact tumor progression, invasion, and metastasis. Apart from immune score, it should be noted that there was no significant discrepancy in the stromal score between the subgroups either in the risk model or in COL3A1 expression (Figures 2B and 3D). This phenomenon reminded us that the risk model and COL3A1 alone have fallen short of presenting the real stromal status in the tumor microenvironment. Despite these limitations, our findings will inform on HNSCC treatment.

## AUTHOR CONTRIBUTIONS

XTY: Conceptualization, Supervision, Writing – review & editing; YCS: Investigation, Writing – original draft; XYL: Data curation, Formal analysis; XDF: Funding acquisition; DMW: Methodology; LXS: Project administration; LMZ: Validation; XL: Visualization.

## FUNDING INFORMATION

This study was funded by the National Natural Science Foundation of China (No. 81871458), the Health Clinical Research Project of Shanghai Municipal Health Commission (No. 202040328), Clinical Research Program of Ninth People's Hospital, Shanghai Jiao Tong University School of Medicine (No. JYLJ201801, JYLJ201911, JYLJ202111) and the China Postdoctoral Science Foundation (No. 2017M611585), Fundamental research program funding of Ninth People's Hospital affiliated to Shanghai Jiao Tong University School of Medicine (No. JYZZ076).

## CONFLICT OF INTEREST

The authors declared that they have no conflict of interest.

## ETHICS STATEMENT

This study was approved by the Human Research Ethics Committee of the Ninth People's Hospital, Shanghai JiaoTong University School of Medicine (Shanghai, China).

## PATIENT CONSENT STATEMENT

Given the retrospective nature of this study, informed consent was waived.

## Supporting information


Figure S1‐S2

Table S1‐S3

## Data Availability

The data sets used and/or analyzed during the current study are available from the corresponding author on reasonable request.
