# Supplementary material for: COL3A1: Potential prognostic predictor for head and neck cancer based on immune‐microenvironment alternative splicing
Source: Cancer Med. 2022 Aug 29;12(4):4882–94. doi: 10.1002/cam4.5170 (PMC9972170; doi:10.1002/cam4.5170)
Supplement: Supplementary file 1 — Figure S1‐S2 [file CAM4-12-4882-s001.docx]

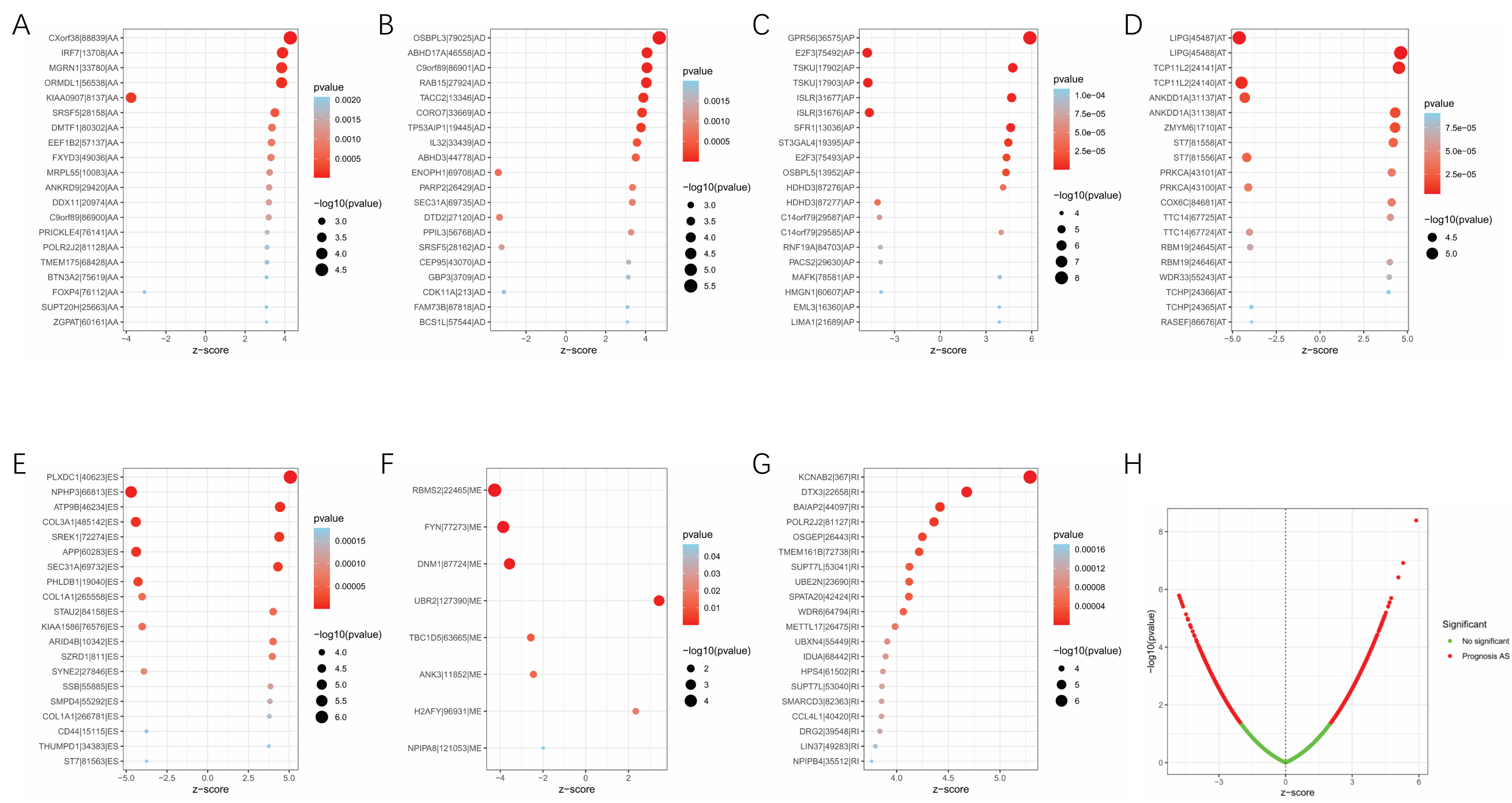


**Fig. S1 The top survival-related AS events in HNSCC.** (A–G) The bubble chart of the top 20 survival-related AS events with ES, AA, AD, AP, AT, RI and top 8 for ME. (H) Overall AS events that are correlated with HNSCC patients’ prognosis.


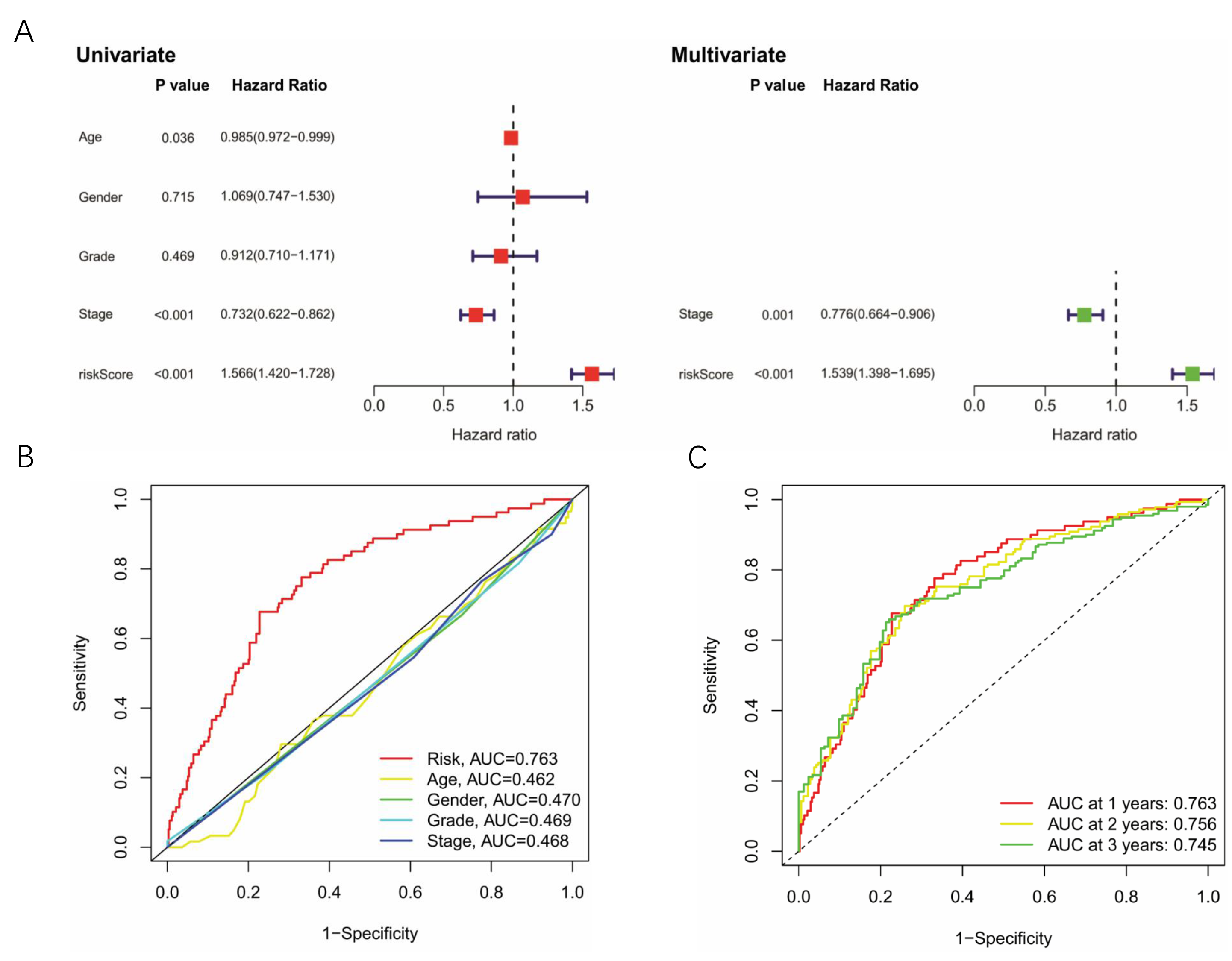


**Fig. S2 Univariate and multivariate analyses and ROC curves.** (A) Univariate cox analysis and multivariate Cox analysis among patients’ age, gender, tumor grade, clinical stage and risk score. (B) ROC curves for patients’ clinical characteristics. (C) ROC curves for risk score at 1, 2, 3 years.
