# Supplementary material for: COL3A1: Potential prognostic predictor for head and neck cancer based on immune‐microenvironment alternative splicing
Source: Cancer Med. 2022 Aug 29;12(4):4882–94. doi: 10.1002/cam4.5170 (PMC9972170; doi:10.1002/cam4.5170)
Supplement: Supplementary file 2 — Table S1‐S3 [file CAM4-12-4882-s002.docx]

**Table S1 Primers for qRT-PCR analysis.**

| COL3A1 Forward | 5’- GCCAAATATGTGTCTGTGACTCA -3’ |
| --- | --- |
| COL3A1 Reverse | 5’- GGGCGAGTAGGAGCAGTTG -3’ |
| GAPDH Forward | 5ʹ- TGACAACTTTGGTATCGTGGAAGG -3ʹ |
| GAPDH Reverse | 5ʹ- AGGCAGGGATGATGTTCTGGAGAG -3ʹ |

**Table S2 A total of 46 immune checkpoint genes.**

| **Genes** | **Full-names** |
| --- | --- |
| TNFSF4 | TNF Superfamily Member 4 |
| CD70 | Tumor Necrosis Factor Ligand Superfamily Member 7 |
| TNFRSF18 | TNF Receptor Superfamily Member 18 |
| TNFSF9 | TNF Superfamily Member 9 |
| LAIR1 | Leukocyte Associated Immunoglobulin Like Receptor 1 |
| KIR3DL1 | Killer Cell Immunoglobulin-Like Receptor 3DL1 |
| TMIGD2 | Transmembrane And Immunoglobulin Domain Containing 2 |
| HHLA2 | HERV-H LTR-Associating 2 |
| CD200 | Antigen Identified By Monoclonal Antibody MRC OX-2 |
| CD48 | Signaling Lymphocytic Activation Molecule 2 |
| TNFSF18 | TNF Superfamily Member 18 |
| CD27 | Tumor Necrosis Factor Receptor Superfamily, Member 7 |
| PDCD1 | Programmed Cell Death 1 |
| CD28 | T-Cell-Specific Surface Glycoprotein |
| IDO2 | Indoleamine 2,3-Dioxygenase 2 |
| CD80 | B-Lymphocyte Activation Antigen B7 |
| TNFRSF8 | TNF Receptor Superfamily Member 8 |
| CTLA4 | Cytotoxic T-Lymphocyte Associated Protein 4 |
| TNFSF15 | TNF Superfamily Member 15 |
| ADORA2A | Adenosine A2a Receptor |
| TNFSF14 | TNF Superfamily Member 14 |
| CD244 | Natural Killer Cell Receptor 2B4 |
| CD40LG | Tumor Necrosis Factor Ligand Superfamily Member 5 |
| TNFRSF9 | TNF Receptor Superfamily Member 9 |
| NRP1 | Neuropilin 1 |
| TNFRSF25 | TNF Receptor Superfamily Member 25 |
| CD276 | B7 Homolog 3 |
| CD40 | Tumor Necrosis Factor Receptor Superfamily Member 5 |
| TIGIT | T Cell Immunoreceptor With Ig And ITIM Domains |
| LGALS9 | Galectin 9 |
| CD160 | Natural Killer Cell Receptor BY55 |
| PDCD1LG2 | Programmed Cell Death 1 Ligand 2 |
| TNFRSF4 | TNF Receptor Superfamily Member 4 |
| CD274 | Programmed Cell Death 1 Ligand 1 |
| LAG3 | Lymphocyte Activating 3 |
| CD86 | B-Lymphocyte Activation Antigen B7-2 |
| BTLA | B And T Lymphocyte Associated |
| ICOS | Inducible T Cell Costimulator |
| IDO1 | Indoleamine 2,3-Dioxygenase 1 |
| HAVCR2 | Hepatitis A Virus Cellular Receptor 2 |
| TNFRSF14 | TNF Receptor Superfamily Member 14 |
| CD44 | Extracellular Matrix Receptor III |
| ICOSLG | Inducible T Cell Costimulator Ligand |
| CD200R1 | CD200 Cell Surface Glycoprotein Receptor |
| VTCN1 | V-Set Domain Containing T Cell Activation Inhibitor 1 |
| BTNL2 | Butyrophilin Like 2 |

**Table S3 The ten basic genes of the risk score formula.**

| **Genes** | **Full-names** |
| --- | --- |
| GPR56 | Adhesion G Protein-Coupled Receptor G1 |
| KCNAB2 | Potassium Voltage-Gated Channel Subfamily A Regulatory Beta Subunit 2 |
| E2F3 | E2F Transcription Factor 3 |
| OSBPL3 | Oxysterol Binding Protein Like 3 |
| ISLR | Immunoglobulin Superfamily Containing Leucine Rich Repeat |
| SFR1 | SWI5 Dependent Homologous Recombination Repair Protein 1 |
| LIPG | Endothelial Cell-Derived Lipase |
| ATP9B | ATPase Phospholipid Transporting 9B |
| BAIAP2 | BAR/IMD Domain Containing Adaptor Protein 2 |
| COL3A1 | Collagen Type III Alpha 1 Chain |
